# Disruptive behaviours involving radiographers that impede a safe work environment. Survey at central hospitals in Harare Metropolitan Province, Zimbabwe

**DOI:** 10.4314/ahs.v22i4.78

**Published:** 2022-12

**Authors:** Bornface Chinene, Busisiwe Pauline Nkosi, Maureen Nokuthula Sibiya

**Affiliations:** 1 Department of Radiography, Harare Institute of Technology, Harare 263, Zimbabwe; 2 Faculty of Health Sciences, Durban University of Technology, 7 Ritson Rd., Musgrave, Berea, Durban 4001, South Africa; 3 Division of Research, Innovation and Engagement, Mangosuthu University of Technology, Jacobs 4026, South Africa

**Keywords:** Disruptive behaviour, radiographers, Zimbabwe

## Abstract

**Background:**

Understanding disruptive behaviours from the perspective of radiographers is important, as this professional group uses hazardous radiation in the execution of their duties, making patient safety of utmost concern.

**Objective:**

Determine the disruptive behaviours involving radiographers at central hospitals in Harare Metropolitan Province, Zimbabwe.

**Methods:**

A descriptive cross-sectional quantitative study was carried out at central hospitals in Harare Metropolitan Province, Zimbabwe, where 100 radiographers were randomly selected.

**Results:**

Overall, 83% of radiographers had been exposed to an incident of DB in the preceding 12 months. Reported types of disruptive behaviour included: Verbal abuse (81%), sexual abuse (21%) and physical abuse (4%). Of the 21 radiographers that suffered sexual abuse, the majority 71 % (n=15) were female while 29% (n=6) were males. Prevalence odds ratio revealed that female radiographers were 1.8 times more likely than their male counterparts to be victims of the workplace sexual abuse (95% C.I.: 0 – 3.04). A significant 69% were abused by patients and their families/escorts, p=.001.

**Conclusion:**

More than 8 out of 10 radiographers in this study were exposed to disruptive behaviours, mostly from the patients and patient's family or escorts. A framework to increases awareness and address these behaviours is recommended.

## Introduction and background

Healthcare workers experience 5–12 times estimated rates of disruptive behaviours (DBs) compared to workers overall^1^. These behaviours have become an unprecedented global problem transcending borders, work settings and professional groups. DBs reported by staff in 98% of healthcare work settings^2^, undermine the rights of patients to safe healthcare and rights of healthcare workers to a healthy work environment^3^. Furthermore, organizational outcomes such as cost, staff turnover, and job satisfaction are also affected^4^. Concerns about DB impact on patient safety led numerous international medical organisations, the Joint Commission standard connected to inappropriate and disruptive behaviour and other healthcare professions to escalate the urgency of knowing the prevalence, causes and consequences of these negative behaviours in different healthcare settings^5^–^8^.

DB is a concept that articulates human behaviour, the work performance in healthcare and patient safety^9^. A series of recent studies has indicated that exposure to DBs can adversely affect the mental abilities required for effective diagnostic and medical performance by healthcare workers^10^–^13^. Furthermore, exposure to DBs hampers the very collaborative mechanisms recognized as essential for patient care and safety^14^. Accordingly, procedural performance of safety protocols by radiographers can be affected by exposure to these behaviours^11^. For example, DBs may cause radiographer confusion, leading to errors in radiation exposure selection, imaging the wrong patient or carrying out the wrong examination. This results in unnecessarily high radiation doses to the patient, jeopardizing patient radiation protection and safety^15^,^16^. The challenge of DBs is therefore, of significant concern for radiographers because they use radiation which has hazardous effects to the living organism cells^17^. Indeed, general literature shows that the implementation of radiation protection and safety practices in radiography has always been done from a technical point of view^18^. The behavioural or humanistic factors in patient safety have been largely ignored^19^. However, the technical point of view does not answer all questions related to radiography practice, in particular the “human” side of the profession, involving the patient encounter and staff working interactions^20^.

Large scale studies done in the developed world, have mainly focussed on the perspective of nurses and physicians^21^, ^22^. Although, DBs are universal in healthcare, in low resource setting radiography there are unique DBs triggers^3^, ^23^, ^24^. There are, however, a few studies exploring DBs in radiography more so, in low resource settings like Africa^25^–^27^. Indeed, according to our knowledge there is no study that evaluates DBs in Zimbabwe and there is no written policy to monitor and prevent DBs in the Zimbabwean radiography workforce. While research findings in other healthcare professions and high resource settings have documented workplace behaviours that undermine patient safety^4^,^28^,^29^, this study focuses on DBs involving radiographers in low resource settings. Understanding the viewpoints of radiographers in these settings is important, as this professional group uses hazardous radiation in the execution of their duties with inadequate resources, making patient safety of utmost concern.

Failure by organisations to gather data on the prevalence, causes and consequences of DBs involving healthcare workers and to provide policy-makers with evidence-based information is tantamount to inability to address the problem8. The purpose of this descriptive cross-sectional quantitative study is to evaluate the DBs that impede a safe radiography work environment at central hospitals in Harare Metropolitan Province (HMP), Zimbabwe. The findings could inform policy development on addressing these behaviours and hence improve patient radiation protection and safety. Additionally, the results could also serve as baseline information for further large-scale studies to closely examine the problem of DBs in the Zimbabwean healthcare labour workforce.

## Methods

### Study design and setting

A descriptive cross-sectional quantitative study was carried out from January to March 2021 at central hospitals in HMP, Zimbabwe, where 100 radiographers were selected.

### Sampling procedures and Sample size for hospitals in HMP

Sampling of the three hospitals in HMP in this study was achieved by criterion purposive sampling, in this case the criteria for selection being a referral hospital in the public sector. This method of sampling was chosen because the aim of the study was to examine DBs in the public sector. Public sector hospitals appear particularly susceptible to incidents of DBs due to increased levels of overcrowding, long waiting times plus staff shortages, unavailability of beds and resources, fewer resources for training and human resources improvement, budget cuts and old or insufficient equipment among other factors^1^, ^25^.

### Sampling procedures and sample size for radiographers in HMP

A total of 100 radiographers registered with the Allied Health Practitioners Council of Zimbabwe, and who had at least one year work experience were included in the study. After the radiographers were identified with the help of Human Resources in each central hospital in HMP, all radiographers who fulfilled the inclusion criteria were identified. The participants' names were then put in a box and randomly selected according the minimum sample for each hospital.

### Study tool and procedure of data collection

Participants were then given a letter of information about the study and those that agreed to participate in the study and were asked to sign a letter of consent. A self-administered questionnaire comprising both closed and open-ended questions was used to collect statistical data from radiographers that satisfied the inclusion criteria. The questionnaire contained a total of 13 items, in order to obtain maximum data for minimum burden on radiographers.

### Data analysis

Descriptive statistics including means and standard deviations, where calculated. Frequencies were represented in the form of tables, graphs and pie charts. In order to test for significant trends in the data, inferential statistics were applied. Throughout a p-value of 0.05 was used to indicate the significance level at 95%. The analysis was carried out using the latest version of the Statistical Package for the Social Sciences (SPSS version 27.0).

### Ethical clearance

To warrant that ethics were upheld, letters of approval were sought from the Durban University of Technology IREC, Ministry of Health and Child Care and the Harare province district administrators. Permissions were also requested from the Medical Research Council of Zimbabwe (MRCZ/A/2684), Parirenyatwa Group of hospitals, Harare Central Hospital (HCHEC081020/47) and Chitungwiza Central Hospital clinical directors respectively. Ultimately, full approval with IREC number 097/20 was granted.

## Results

### Demographics of the participants

A total of 100 (n=100) radiographers working at the three central hospitals in HMP, participated in the survey. The sample consisted of 56 female (56%) and 44 male (44%) radiographers all at least 21 years old. Most of the radiographers (70%) were in the age group 21–30, 25% were in the age group 31–40 and only 5% were above 40. In terms of marital status, the majority of radiographers were single (72%), 24% were married and the remainder were either divorced (4%) or widowed (1%). Regarding academic qualifications, 87% had a Bachelor's degree, 10% were holders of a master's degree and only 3% had a diploma as the highest qualification. Most of the radiographers (53%) in the sample were employed at Hospital A. The radiographers were drawn from the three radiography departments namely radiology, radiotherapy and the school of radiography which incorporates Nuclear Medicine. The other 28% was from Hospital B and 19% from Hospital C both of which have radiology departments only. A large proportion of the radiographers (66%) had less than 5 years' work experience, 18% had 5–10 years' work experience and 6% had over 15 years work experience. In terms of the grade, 65% were basic radiographers, 21% were senior radiographers, 9% principal grade and 5% were chief radiographers. Table 1 below summarises the participant's demographics.

**Table 1:** Summary of socio-demographic and professional characteristics of respondents

Characteristic	Number *(n)*	Percentage *(%)*
**Gender**		
Male	44	44.0
Female	56	56.0
**Age group**		
21–30	70	70.0
31–40	25	25.0
40 and above	5	5.0
**Marital Status**		
Single	72	72.0
Married	24	24.0
Divorced/Separated	3	3.0
Widowed	1	1.0
**Work experience (Years)**		
Below 5	66	66.0
5–9	18	18.0
10–1515 and above	10	10.0
	6	6.0
**Academic qualification**		
Diploma	3	3.0
Bachelor's degree	87	87.0
Master's Degree	10	10.0
**Grade**		
Basic radiographer	65	65.0
Senior radiographer	21	21.0
Principal radiographer	9	9.0
Chief radiographer	5	5.0

### Prevalence of DBs involving radiographers in HMP

A significant 61% of the radiographers indicated that they had been exposed more than once, (χ2 (2) = 34.820, p<.0005). In addition, 22% had been exposed only once and 17% indicated that they had not been exposed to a single incident of DB in the past year prior to the study. This, therefore, gives an overall prevalence of 83% at the time of the study. When asked if they had ever witnessed a radiographer being exposed to a DB incident in their current workplace in the past 12 months, a significant 74% of radiographers in HMP said they had indeed witnessed at least one, (χ2 (1) = 23.040, p<.0005).

### Types of DBs involving radiographers in HMP

Most of the radiographers reported verbal abuse, followed by sexual abuse and then physical abuse. A significant 81% had been exposed to verbal abuse, (χ2 (1) = 45.375, p<.0005). A further 21% were exposed to sexual abuse, (χ2 (1) = 24.045, p<.0005). Lastly, 4% were exposed to physical abuse, χ2 (1) = 73.719, p<.0005). Table 3 below summarises the prevalence of the different types of abuses suffered by radiographers. There was no statistically significant association between hospitals and being verbally abused. Additionally, age was not significantly associated with being verbally abused.

**Table 3:** Prevalence of the different types of DBs suffered by radiographers

Exposure to DBs	Verbal abuse %	Physical abuse %	Sexual abuse %
Yes	81	4	21
No	15	85	67
Missing	4	11	12
Total (%)	100	100	100

A total of 21 radiographers suffered sexual abuse, the majority 71 % (n=15) were female while 29% (n=6) were males. A calculation of the prevalence odds ratio revealed that female radiographers were 1.8 times more likely than their male counterparts to be victims of the workplace sexual abuse (95% C.I.: 0 – 3.04). On the other hand, the bivariate analysis showed that a significant number of males said they had NOT been verbally abused, p=.012 compared to women. Additionally, a significant number of females had been abused by a fellow radiographer, p=.015, while a significant number of males indicated that they had been abused by a doctor, p=.032

### Perpetrators of DB s involving radiographers

**Figure 1:**
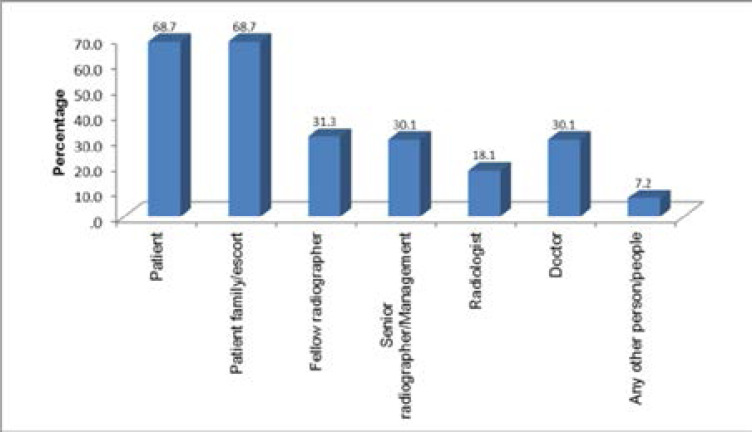
Perpetrators of DBs involving radiographers

Radiographers who had experienced DBs in their workplace were asked to state the perpetrators (Figure 1). The respondents described perpetrators of DBs as mostly patients and their families/escorts. A significant 69% had been abused by a family member or escort of a patient, p=.001. Radiographers abused by fellow radiographers, senior management and doctors, were 31.3%, 30.1% and 30.1% respectively. Those that were abused by any other, most mentioned nurses (n=5) and security guards (n=2) as the culprits.

## Discussion

To the researcher's knowledge, this is the first study to document DBs involving radiographers in Zimbabwe. The findings reveal an expected high prevalence (83%) of exposure within 12 months prior to the study. These findings suggest that the majority of radiographers at central hospitals in HMP are suffering from these perilous incidents in their workplaces. Regardless of some differences in the definition of these behaviours, targeted healthcare professional groups, methods employed and sample size, the prevalence of DBs in this study is comparable to and higher than in most studies in the literature as shown in Table 4 below 24,27,31–34.

**Table 4:** Prevalence and types of disruptive behaviours among health professionals in the literature

DBs	Zimbabwe*	Gambia (27)	Namibia (24)	Egypt (31)	Hong Kong (32)	South Africa (33)	USA (34)
**Prevalence** (%)	83	62.1	100	79.8	61	-	51.7
**Verbal** **abuse (%)**	81	59.8	100	98.7	96.7	73	-
**Sexual** **abuse (%)**	21	10	84.6	1.3	10.3	-	-
**Physical** **abuse (%)**	4	17.2	46.2	38.7	20.8	14	-

* Current study

Most studies, however, have evaluated the prevalence of DBs in either the developed world and/or have mainly focused on the perspective of nurses or physicians^35^, ^36^. This study is significant because, in spite of, DBs being universal in healthcare, each healthcare profession and setting presents unique DB triggers^3^, ^23^. Findings of this study can serve as baseline information for further studies to closely examine the problem of DBs in the Zimbabwean radiography labour workforce. By extension, the results may also contribute to existing literature in the low resource settings where the topic is under-researched^26^, ^27^.

### Types of DBs

• **Verbal abuse**

The most common type of DBs reported in this study was verbal abuse, and this is consistent with the literature, as shown by Table 8.1 above. These findings were anticipated because it is difficult to pinpoint verbal abuse, and to get the aggressor penalized as is because people with different personalities have different levels of tolerance for gossip, teasing or sexual jokes.

• **Sexual and physical abuse**

However, sexual abuse was the second-highest while physical abuse was the least common in the present study. This was at odds with most studies (Table 4), but similar to the quantitative study done by Hattingh et al.^24^, in a single centre in Namibia. Despite the small sample size (13 radiographers) in their study, Hattingh et al. attributed this to a lack of awareness of what constitutes sexual harassment among the perpetrators and the radiographers. Nevertheless, the current study attributes the high prevalence of sexual abuse to other additional factors including:

*Gender hegemony* - Socio-cultural factors such as patriarchal gender affairs may influence healthcare workplace behaviours. In this study, female radiographers were more likely to be victims of the workplace sexual abuse. In the Shona culture, patriarchal practices shape and propagate gender hegemonic norms in the workplace because according to Kambarami^37^, “custom in Africa is stronger than domination, stronger than the law, stronger even than religion”. Indeed, a study done by the Transparency International Zimbabwe^38^, concluded that sexual harassment in Zimbabwe is institutionalised, and that women have been suffering for a very long time.

*Lack of mechanisms to detect or identify sexual harassment and mechanisms to support victims of sexual abuse* – The Zimbabwean Constitution (No 20 of 2013) does not explicitly provide for protection against abuse, there is no definition of sexual harassment in the Labour Act (28:01), and the Public Service Act (16:4) does not include disciplinary procedure for sexual harassment. Nonetheless, Statutory Instrument 1 of 2000 tersely includes sexual harassment as a mere misconduct^39^. It is, therefore, conceivable that perpetrators may be taking advantage of these factors to pounce on women mainly in the different workplaces. In conclusion, the results suggest that healthcare leaders must institute additional policies to combat sexual abuse in their workplaces^38^. The latter discussion reveals how by-laws can influence workplace behaviour; however, more research is required.

• **Perpetrators of DBs**

Patients and their escorts or family members were the main perpetrators of the DBs in this study. This was congruent with findings from previous studies^24^, ^32^. According to Vogel^40^, traditionally, patients are seldom charged abuse on healthcare workers because they are not in control of their faculties when compromised by illness, distress or drugs. Consequently, radiographers endure the patients' abuse in HMP. In the developed world, healthcare workers are canvassing for harsher legal penalties for abusive patients^40^. The current study found that DBs can also be committed by radiographers themselves or any member of the healthcare team. This was also reported in previous studies 24, 31, 33.

## Limitations and recommendations

Resource constraints and time limited participation to only radiographers at central hospitals in HMP, Zimbabwe. Consequently, these results may not be generalized to the private and any other groups of public healthcare institutions in Zimbabwe. Based on the findings, we recommend a qualitative study that further explores the experiences of radiographers exposed to incidents of DBs and how they affect patient radiation protection and safety.

## Conclusions

More than 8 out of 10 radiographers in this study were exposed to DBs, suggesting that the majority of radiographers at central hospitals in HMP are suffering from these perilous incidents in their workplaces. A framework to improve awareness and address these behaviours is therefore recommended to promote healthy work environments that permit radiographers to focus on delivering superior, affordable, and safe patient care.

## Figures and Tables

**Table 2:** Summary of radiographers exposed to incidents of DBs (n=100)

Exposure to DBs	Percentage (%)	Cumulative percentage (%)
**Yes**		
Once	22	22
More than once	61	83
**Not at all**	17	100
